# Potential health benefits of blueberry and raspberry pomace as functional food ingredients: Dietetic intervention study on healthy women volunteers

**DOI:** 10.3389/fnut.2022.969996

**Published:** 2022-08-17

**Authors:** Tamara Popović, Bojana Šarić, Jasmina Debeljak Martačić, Aleksandra Arsić, Pavle Jovanov, Edita Stokić, Aleksandra Mišan, Anamarija Mandić

**Affiliations:** ^1^Department in Food and Metabolism, Institute for Medical Research University of Belgrade, Belgrade, Serbia; ^2^Institute of Food Technology, University of Novi Sad, Novi Sad, Serbia; ^3^Diabetes and Metabolic Disorders, Department of Endocrinology, Medical Faculty, University of Novi Sad, Novi Sad, Serbia

**Keywords:** dietetic intervention study, berry pomace, metabolic disorder, lipid status, plasma fatty acids

## Abstract

The fruit juice industry generates pomace as a valuable by-product especially rich in polyphenols, dietary fibers, vitamins, minerals, and unsaturated fatty acids. In the cookies used in this study, 30% of the gluten-free flour was replaced with dried and ground blueberry and raspberry pomace, rich source of polyphenols, dietary fibers, linoleic and alpha-linolenic acid. In order to examine whether the addition of blueberry and raspberry pomace in cookie formulation can have beneficial effects on certain blood parameters and anthropometric measurements, the designed cookies were tested in 20 healthy, normally fed female subjects, aged 30–50 years (41.35 ± 8.58 years) over four-week dietetic intervention study. Significant changes in the composition of fatty acids serum phospholipids, decrease in LDL-cholesterol level (20.16%), increase in adiponectin level (25.52%) and decrease in ALT and AST values were observed, thus indicating that inclusion of cookies containing blueberry and raspberry dried and ground pomace to usual diet might have positive effects on certain cardiovascular risk factors and liver function indicators.

## Introduction

During the juice processing, a large quantity of valuable bioactive compounds remains in the pomace. According to the study of Khanal et al. (1) more than 50% of proanthocyanidins from fresh blueberry remain in skin and pulp residue after juice production. Raspberry pomace was found to be rich source of essential fatty acids, dietary fibers and antioxidants (2, 3) since it consists mainly of seeds (80%) and pulp (4, 5). Most of the studies regarding blueberry and raspberry pomace as potential low-cost food ingredients are mainly focused on the identification of valuable bioactive compounds (6–15). Only few studies investigated their application in different food systems and the effects on the products' nutritional composition, physico-chemical, technological, and sensory properties (16–20).

Concerning the health-promoting effects of bioactive compounds from blueberry and raspberry, this dietetic intervention (pilot) study was organized to investigate the potential health effects of blueberry and raspberry pomace in concentrated (dried and ground) form, incorporated in food system. Cookies were included in daily diet, to evaluate the effect of blueberry and raspberry on certain factors strongly correlated with cardiometabolic risk (lipid status, fasting glucose, and adiponectin level), as well as on biomarkers of hepatic and renal function and anthropometric measurements. To the best of the author's knowledge, this is the first dietetic intervention study that investigates the potential health benefits of bioactive compounds from blueberry and raspberry pomace incorporated into the food system. These ingredients were chosen to be incorporated into gluten-free cookies to design the functional food product primarily intended for the population with coeliac disease, but also suitable for the healthy population.

## Materials and methods

### Dietetic intervention study

The dietetic intervention study was conducted according to the Declaration of Helsinki and was approved by the Ethics Committee of the Clinical Center of Vojvodina, Novi Sad, Serbia (approval number 00-01/825/2011). Women of reproductive age were selected for the study, considering the possible effect of sex hormones on the level of lipids and lipoprotein status. Also, following the results of studies that examined the therapeutic efficacy of statins in reduction of CV risk in primary and secondary prevention and showing the dependence on gender, we included only female subjects in the study. The study included 20 healthy women, aged 30–50 years (41.35 ± 8.58 years), with no pre-existing any active or chronic disease, with no plan to alter eating and exercise habits for the duration of the study. Smokers, subjects in acute stress situations or with infections, as well as those with recent (i.e., 3 months) weight changes and any supplementation with micronutrients and hormonal contraception, as well as pregnant or menopausal women, patients with hepatic, psychiatric or malignant disorders were excluded from the study. During the 4 weeks study, all participants consumed four cookies (32 g) per day without a change in dietary habits. Control group was not formed because the aim was to measure significance of changes before and after treatment, and not to measure therapeutic efficiency. The study was organized as a pilot study, as a small scale preliminary study. The cookies were produced according to the procedure given in Šarić et al. (18), packed in polypropylene bags and distributed to participants at the beginning of the study. The content of macronutrients (proteins, carbohydrate, fats, dietary fibers, essential fatty acids–linoleic and α-linolenic) as well as antioxidant properties of cookies used in this study were previously reported by Šarić et al. (18).

### Anthropometric measurements and biochemical analysis

Body weight (BW, kg) and body height (BH, m) were measured and body mass index was calculated as BW/BH^2^ (kg/m^2^). Body weight was measured using a balanced beam scale, to the nearest 0.1 kg. Body height was measured using a Harpenden anthropometer (Holtain Ltd, Croswell, UK), to the nearest 0.1 cm.

Blood samples were collected prior the intervention study (baseline) and at the end of the study, after 12 h fasting period, into sample tubes for serum and tubes with ethylenediaminetetraacetic acid (EDTA) as an anticoagulant for plasma. Biochemical parameters were determined from serum on the day of samples collection. Samples from EDTA tubes were centrifuged (1800 × g, 10 min), for the separation of plasma, and aliquots were stored at −80°C for further analyses.

Serum levels of triglycerides, total cholesterol, HDL, LDL, creatinine, urea, and uric acid were measured using the standard biochemical methods. All biochemical parameters were determined using Hitachi 912 automatic bioanalyser (Roche Diagnostic GmbH, Mannheim, Germany) with reagents from Analyticon Biotechnologies AG (Lichtenfels, Germany). Total cholesterol was determined by the enzymatic colorimetric CHOD-PAP method, triglycerides by the GPO-PAP method, HDL cholesterol by an enzymatic colorimetric direct HDL assay, and low density lipoprotein (LDL) by a homogeneous enzymatic direct LDL assay. Fasting plasma glucose was determined using GOD-PAP (glucose oxidase – phenol and aminophenazone) method. Creatinine was determined according to Jaffe reaction kinetic method using alkaline picrate reagent, while a blood urea nitrogen by a kinetic method using the coupled urease/glutamate dehydrogenase (GLDH) enzyme system. The level of uric acid in the blood was determined using modified PAP method.

### Fatty acid analysis of plasma lipids

Total lipids in plasma were extracted with a mixture of chloroform/methanol (2:1, v/v) by a slightly modified method of Folch. (19) The lipid classes were separated by thin-layer chromatography (TLC) for detection of phospholipids above other lipids classes. Fatty acid methyl esters (FAMEs) from detected phospholipids were analyzed by gas-liquid chromatography on a Shimadzu chromatograph (GC 2014, Kyoto, Japan) equipped with capillary column (Rtx 2330, 60 m × 0.25 mm internal diameter, the film thickness of 0.2 μm, RESTEK, Bellefonte, PA, USA). The identification of FAMEs was accomplished by comparing the retention times of sample peaks with standard mixture (PUFA-2, Supelco, Bellefonte, PA, USA). (19) Fatty acid content is expressed as a percentage of total identified fatty acids.

### Statistical analysis

Statistical analysis of the data obtained by the dietetic intervention study was performed using the SPSS (version 11.0) software. The results are given as mean values with standard deviations. The normality test was Shapiro-Wilk before statistical analysis. Since all variables showed normal distribution, statistical comparisons of means were performed using the paired Student's t-test. *p* values lower than 0.05 was considered statistically significant.

## Results

This study was performed on normal weight female subjects who included in their diet 32 g of the tested cookies per day, without a change in dietary habits. Anthropometric characteristics and biochemical parameters are shown in Table 1. Significant (*p* < 0.01) reduction of LDL-cholesterol level was observed at the end of study (20.16%). The level of total cholesterol, HDL-cholesterol and triglycerides was not changed significantly, and can be categorized as desirable lipid status, both before and after the study. Fasting and postprandial glucose levels were not significantly changed and they were within the reference range. Parameters relevant to liver function (AST and ALT) were found to be significantly (*p* < 0.05) lower at the end of study, while serum creatinine, urea and uric acid value were not significantly changed. As a parameter related to anti-inflammatory effects which can contribute to prevention of atherosclerosis, adiponectin was determined at the beginning and the end of the study and significant (*p* < 0.05) increase of its concentration was observed (25.52%). However, the highest impact of the cookie consumption on certain blood parameters was noticed during the study for fatty acid profile of serum lipids (Table 2). Significant reduction (*p* < 0.05) in saturated fatty acids (SFA) was observed with the highest decrease in palmitic (16:0) acid concentration (5.88%). On the other hand, the percentage of docosapentaenoic acid (22:5 n-3, DPA) and docosahexaenoic acid (22:6 n-3, DHA) was significantly (*p* < 0.05) increased after the intervention period. Accordingly, the percentage of total n-3 fatty acid significantly (*p* < 0.01) increased, while the ratio of n-6/n-3 significantly (*p* < 0.05) decreased at the end of the study.

**Table 1:** Anthropometric characteristics and biochemical parameters of the study participants at baseline and after 4 week intervention.

**Parameters**	**Baseline**	**End of study**	***p*-value**
Body mass (kg)	65.54 ± 10.26	65.59 ± 10.36	0.9676
BMI (kg/m^2^)	23.10 ± 3.58	23.12 ± 3.59	0.9246
Fasting glucose (mmol/L)	4.65 ± 0.35	4.65 ± 0.38	0.9031
Postprandial glucose (mmol/L)	4.57 ± 0.43	4.58 ± 0.45	0.9892
Triglycerides (mmol/L)	1.07 ± 0.55	0.96 ±0.41	0.519
TC (mmol/L)	5.99 ± 1.05	5.65 ± 0.94	0.2976
HDL-C (mmol/L)	1.91 ± 0.48	1.87 ±0.45	0.8283
LDL-C (mmol/L)	3.72 ± 0.99	2.97 ± 0.64	0.0080**
Creatinine (mmol/L)	60.72 ± 6.24	60.61 ± 6.42	0.7553
Urea (mmol/L)	4.01 ± 1.19	3.64 ± 1.22	0.2914
Uric acid (nmol/L)	232.49 ± 38.19	234.47 ± 37.21	0.9138
AST (IU/L)	13.89 ± 3.32	11.67 ± 3.13	0.0424*
ALT (IU/L)	19.78 ± 3.97	17.37 ± 4.09	0.0133**
Adiponectin (mg/mL)	11,99 ± 3,29	15,05 ± 5,47	0,0439*

Values are given as mean ± SD; Significantly different values at the end of study compared to baseline were marked as follows: *p < 0.05, **p < 0.01, ***p < 0.001.

**Table 2:** Fatty acid profile in plasma phospholipids of study participants at baseline and after 4 week intervention.

**FA (%)**	**Baseline**	**End of study**	**p-value**
16:0	32.31 ± 2.27	30.41 ± 2.29	0.00096***
16:1	0.61 ±0.11	0.49 ± 0.13	0.000685***
18:0	17.38 ± 2.26	17.17 ± 2.00	
18:1 n9	8.03 ± 0.94	8.14 ± 1.01	
18:1 n7	1.61 ± 0.28	2.28 ± 2.83	
18:2	23.61 ± 2.44	24.40 ± 3.17	
18:3 n3	0.21 ± 0.08	0.21 ± 0.09	
20:3	2.52 ± 0.73	2.72 ± 0.70	
20:4	10.59 ± 2.05	10.74 ± 2.65	
20:5	0.31 ± 0.17	0.31 ± 0.15	
22:4	0.40 ± 0.09	0.39 ± 0.10	
22:5	0.41 ± 0.10	0.46 ± 0.10	0.062385*
22:6	2.01 ± 0.64	2.29 ± 0.63	0.019312**
SFA	49.69 ± 2.41	47.58 ± 3.39	0.019643**
MUFA	10.25 ± 1.18	10.91 ± 3.21	
PUFA	40.06 ± 2.72	41.52 ± 2.94	
n6	37.12 ± 2.57	38.25 ± 2.70	
n3	2.94 ± 0.78	3.27 ± 0.76*	0.013905*
n6/n3	13.38 ± 3.27	12.26 ± 2.72*	0.033049*

Values are given as mean ± SD; Significantly different values at the end of study compared to baseline were marked as follows: *p < p < 0.05, **p < p < 0.01, ***p < p < 0.001.

## Discussion

By using blueberry and raspberry pomace in the form of dried powder, a significant concentration of their bioactive compounds was achieved in the cookie formulation. The previous results (18) indicate that the cookies used in this study are exceptional source of dietary fibers (7.83 ± 0.89 g/100 g) and unsaturated fatty acids (2.47 g/100 g). Daily portion of the cookies consumed by participants in this study meets significant percentage of Dietary Reference Intake (DRIs) given by Food and Nutrition Board of the National Research Council for adult female (30–50 years of age) for dietary fibers (9.98%), α-linolenic (21.95%) and linoleic acid (4.95%). These results are especially important concerning the fact that cookies are among the most frequently consumed snack food and they are usually considered as the products with a poor nutritional profile. Although there is no recommended daily intake of polyphenols, these cookies can be considered an exceptional source of these bioactive compounds due to high content of total phenolics and monomeric anthocyanins (462.12 ± 1.81 mg/100g and 246.81 ± 10.11 mg/100 g, respectively) (18).

Concerning the profile of cookies' bioactive compounds, the study was designed to monitor changes in composition of fatty acids serum phospholipids and certain blood parameters of normal weight female subjects who included cookies in their diet without change in dietary habits (21). The evaluation of possible changes in eating/dietary habits was carried out on the basis of anamnestic data - interview with special reference to the characteristics of the diet (frequency, composition) and the level physical activity (intensity, frequency). Significant reduction of serum LDL-cholesterol during 4 week intervention study could be attributed to the synergistic effects of dietary fibers, unsaturated fatty acids and high daily dose of anthocyanins provided by cookie consumption. LDL cholesterol-lowering effects of individual above-mentioned bioactive components are found to be highly dependent on the form in which they are consumed and health status of the participants included in the study. The overall pooled effect provided by meta-analysis (22) showed non-significant reduction of LDL-cholesterol in the subgroup of healthy population being subjected to dietary sources of anthocyanins. Effect of α-linolenic acid on lowering of LDL-cholesterol was also found to be non-significant (23) while the results of LDL-cholesterol lowering effects of dietary fibers are opposite (24). However, combined effects of these bioactives in the tested cookies resulted in reduction of serum LDL-cholesterol.

Significant increase in DPA and DHA after the intervention can be attributed to high daily intake of α-linolenic acid. These fatty acids with documented positive effects on brain function can be obtained directly from the diet or synthesized in the body from α-linolenic acid (ALA) (25). However, DHA synthesis rates from ALA were suggested to be low, and debate exists as to whether the rate of DHA synthesis is sufficient to meet the requirements (26). Therefore, the significant increase in DPA and DHA level after the intervention can be treated as very promising result. Significant decrease in total SFA and palmitic acid in plasma phospholipids after the intervention as well as above mentioned changes in blood parameters can be good predictor for lowering risk of cardiovascular diseases related to cookie consumption.

The level of adiponectin, which is supposed to have an important role in the development of atherosclerosis and insulin resistance, was also determined within the study. Recent studies indicate the inversely proportional relationship of adiponectin concentration in the blood and metabolic syndrome as well as its anti-inflammatory and anti-atherogenic potential in the treatment of metabolic syndrome and type 2 diabetes (27, 28). The increase in adiponectin level observed in this study indicate a potentially protective effect of the cookies. Slight decrease of ALT and AST enzyme activities could be related to slight improvement in liver health due to cookie consumption. Inflammatory parameters, such as CRP, IL, fibrinogen, etc. were not evaluated, because the primary goal was only the measurement of atherogenic LDL cholesterol, as an additional reduction of LDL cholesterol (“the lower-the better”).

Although the results of this pilot dietetic intervention study primarily emphasized the potential health benefits of consumption the cookies with concentrated blueberry and raspberry bioactive compounds, further investigation of the achieved positive effect should to be conducted in a randomized double blind placebo control study which will include coeliac patients of both sexes. Nevertheless, one can find a cookie formulation as an inspiring model for a functional confectionery formulation. Furthermore, the food industry by-products rich in bioactives were used, which is in line with circular bioeconomy demands.

## Data availability statement

The raw data supporting the conclusions of this article will be made available by the authors, without undue reservation.

## Ethics statement

The dietetic intervention study was conducted according to the Declaration of Helsinki and was approved by the Ethics Committee of the Clinical Center of Vojvodina, Novi Sad, Serbia (approval number 00-01/825/2011). The patients/participants provided their written informed consent to participate in this study.

## Author contributions

TP contributed to experimental work, statistical analysis, writing-original draft. BŠ contributed to conception, experimental work, writing-original draft and editing. PJ, JM, and AA contributed to experimental work. ES contributed to experimental work and supervision. AMi and AMa contributed to conception, supervision and writing-review. All authors contributed to the article and approved the submitted version.

## Funding

This work is financed by the Ministry of Education, Science and Technological Development of the Republic of Serbia (TR 31029 and 451-03-68/2022-14/2022) and 451-03-68/2022-14/200015.

## Conflict of interest

The authors declare that the research was conducted in the absence of any commercial or financial relationships that could be construed as a potential conflict of interest.

## Publisher's note

All claims expressed in this article are solely those of the authors and do not necessarily represent those of their affiliated organizations, or those of the publisher, the editors and the reviewers. Any product that may be evaluated in this article, or claim that may be made by its manufacturer, is not guaranteed or endorsed by the publisher.

## Abbreviations

BW: body weight
BH: body height
BMI: body mass index
TC: total cholesterol
HDL-C: high-density lipoprotein cholesterol
LDL-C: low-density lipoprotein cholesterol
AST: alanine aminotransferase
ALT: alanine aminotransferase
FA: fatty acid
SFA, saturated fatty acids, MUFA: monounsaturated fatty acids
PUFA: polyunsaturated fatty acids
DHA: docosahexaenoic acid
DPA: docosapentaenoic acid
FAMES: fatty acid methyl esters
ALA: α-linolenic acid.
